# Establishment and characterization of preclinical models of human gynecologic tract carcinosarcomas demonstrates targetable FGFR1 alterations

**DOI:** 10.1016/j.tranon.2025.102591

**Published:** 2025-11-06

**Authors:** Nelson K.Y. Wong, Marta Llaurado Fernandez, Hannah Kim, Pooja Praveen Kumar, DuPreez Smith, James Key, Yen-Yi Lin, Stanislav Volik, An Nhien Tong, Stephane Le Bihan, Colin C. Collins, Yangxin Fu, Hui Xue, YZ Wang, Martin Köbel, Mark S. Carey, Cheng-Han Lee

**Affiliations:** a Department of Experimental Therapeutics, BC Cancer, Vancouver, British Columbia, Canada; b Department of Obstetrics and Gynecology, University of British Columbia, Vancouver, Canada; c Department of Obstetrics and Gynecology, University of Alberta, Edmonton, Alberta, Canada; d Department of Laboratory Medicine and Pathology, University of Alberta, Edmonton, Alberta, Canada; e Vancouver Prostate Centre, Vancouver, British Columbia, Canada; f Department of Oncology, University of Alberta, Edmonton, Alberta, Canada; g Department of Pathology and Laboratory Medicine, University of Calgary, Calgary, Alberta, Canada; h Department of Laboratory Medicine and Pathology, Royal Alexandra Hospital, Edmonton, Alberta, Canada

**Keywords:** Carcinosarcoma, MMMT, FGFR1, Xenograft tumor

## Abstract

**Objective:**

Gynecologic carcinosarcoma is an uncommon but aggressive malignancy that frequently requires systemic therapy but therapeutic options are limited. Development of preclinical models is therefore important for therapeutic advancement.

**Methods:**

Carcinosarcoma tumor (6 uterine and 1 tubo-ovarian) from 7 surgical samples were implanted into immunocompromised mice for patient-derived xenograft (PDX) and/or cell line development. The histologic, immunophenotypic and genetic features were characterized. Based on the observed molecular profiles and targetable molecular alterations, *in vivo* studies were conducted to evaluate the efficacy of targeted therapy on tumor growth.

**Results:**

We established 1 cell line and 6 PDX models which recapitulated the dominant phenotype of the respective parental tumors with preserved mesenchymal differentiation lineage in the sarcomatous component. Genomically, the PDX/cell line models preserved similar complex pattern of copy number alterations and similar mutation landscape when compared to the respective parental tumors. All 7 parental carcinosarcoma tumors and PDX/cell line models harbored pathogenic *TP53* mutations. Moreover, we identified recurrent copy number gain/amplification involving several receptor tyrosine kinases (RTK), including amplification and protein over-expression of *FGFR1. In vivo* drug evaluation using a small molecule inhibitor (AZD4547) of FGFRs showed significant growth inhibition in the carcinosarcoma PDX tumor with the highest *FGFR1* amplification and protein expression whereas AZD4547 showed no significant growth effects on carcinosarcoma lacking high level *FGFR1* amplification, indicating oncogenic dependency on the amplified RTK pathway.

**Conclusions:**

These findings demonstrate the utility of patient-derived tumor models in the identification and the functional validation of potentially targetable molecular alterations in preclinical setting.

## Introduction

Carcinosarcoma of the gynecologic tract is a clinically aggressive malignant biphasic tumor comprised of a mixture of carcinomatous element and sarcomatous element [[1], [2], [3]]. It most frequently arises in the uterus (endometrium) and more rarely in the ovaries. About 40 % of uterine carcinosarcoma and the majority of ovarian carcinosarcoma present with advanced stage disease that is not amenable to curative surgery. There is limited role for adjuvant radiation therapy and although conventional platinum/taxane-based results in a survival benefit in p53 mutated carcinomas, mortality rates in patients with advanced disease are appreciable [4]. The development of more effective systemic therapy options is therefore urgently needed.

Over the past two decades, we have gained significant insight into the molecular basis of gynecologic (Mullerian) carcinosarcoma. Genomic analyses have shown that the carcinomatous and the sarcomatous components are clonally related and that the mutation profiles resemble those of the carcinoma from the respective anatomic sites [[5], [6], [7], [8], [9]]. Almost all endometrial carcinosarcomas, show a *TP53*-mutated/copy number-high molecular profile based on the TCGA (The Cancer Genome Atlas)-derived molecular classification for endometrial carcinoma, while a few may exhibit copy number-low/no-special-molecular profile (NSMP), microsatellite instable (MSI-H)/mismatch repair protein-deficient (MMRd) or *POLE*-mutated molecular profiles [5,6,8,10,11]. These studies suggest that carcinosarcomas arise through sarcomatous transdifferentiation of a mullerian carcinoma. However, despite ongoing sequencing efforts, the molecular trigger(s) underlying the sarcomatous transdifferentiation have not been identified to date. There is also no apparent directionality for tumor progression based on the mutation load, as the carcinomatous component harbors greater additional somatic mutations compared to the sarcomatous component in some cases and *vice versa* in other cases [5,6]. It thus appears that sarcomatous transdifferentiation in gynecologic carcinosarcoma may represent a phenotypic marker for a disrupted state of cellular lineage control of the underlying mullerian carcinoma, which is associated with more aggressive clinical behavior.

As gynecologic carcinosarcomas are a rare cancer, clinical trial capacity is limited. Therefore, the creation of clinically representative experimental models for preclinical drug evaluation and prioritization is critical for successful therapeutic development. Patient-derived xenografts (PDX) are valuable research tools that can capture the complexity of the parental tumour, particularly in a tumor type such as carcinosarcoma that lack engineered animal models. Moreover, when scaled up, PDX models can also captured and reflect the molecular diversity that exists in carcinosarcoma. As there are currently only a limited number of molecularly annotated PDX models of uterine and ovarian carcinosarcoma reported to date [[12], [13], [14], [15], [16]], the purpose of this study is to develop additional PDX models of gynecologic carcinosarcoma with comprehensive histologic, immunophenotypic and genomic characterization/validation to expand the molecular diversity of PDX models for therapeutic development. We report here the successful establishment of 5 uterine carcinosarcoma and 1 ovarian carcinosarcoma PDX models that recapitulate the phenotypic and molecular characteristics of the parental tumors, with the identification of potentially targetable molecular alterations for therapeutic evaluation.

## Materials and methods

### Patient samples and collection

Patients undergoing surgical resection at Vancouver General Hospital (Vancouver, Canada) with a pre-operative biopsy-proven or intra-operative frozen section-proven diagnosis of gynecologic tract carcinosarcoma (treatment-naive) and with consent for tissue collection were included in the study. Over a two-year study period (2017–18), a total of 8 gynecologic tract carcinosarcomas with sufficient grossly apparent tumor tissue available for research collection were included for the development of patient-derived xenograft (PDX) and cell line tumor model development. Resected tumor was expediently transported to pathology laboratory to minimize ischemic time (less than 15 min) and the fresh tissue was harvested immediately with a portion transported to research laboratory as well as cryopreserved in 90 % FBS/10 % dimethyl sulfoxide for PDX model development and a portion dedicated for immediate cell culture. Additional adjacent tumor tissue was collected, with a portion submitted for formalin fixation and a portion snap frozen for further molecular analyses; peripheral blood from corresponding patient was also collected. The entire study including patient consent, tumor collection, PDX model and cell line model development was approved by institutional research board (H19–02,823, H17–01,863 and A18–0105).

### Patient-derived xenograft (PDX) development

All PDX models were initially implanted under the subrenal capsule of NOD-*scid* IL2Rgamma^null^ or NOD-*Rag1^null^ IL2rg^null^* mice (1–2/kidney) to optimize the success rate of tumor engraftment as previously described with modifications and in the Supplementary Materials and Methods [17]. The PDXs were serially passaged in the subrenal site to passage #3, at which time the PDX tumor line was considered stable. The PDXs were then transferred to subcutaneous location and further passaged.

### Establishment and maintenance of a patient-derived carcinosarcoma cell line

A patient-derived cell lines was established in-house through continuous *in vitro* culture of primary patient material (tumor tissue). Carcinosarcoma cells were established and maintained in M199:MCDB105 (1:1) media (Cat. No. M5017, Cat. No. M6395, Sigma-Aldrich, Oakville, Ontario, Canada) supplemented with 10 % defined fetal bovine serum (dFBS; Cat. No. SH30070.03, Hyclone, GE Life Sciences, Logan, UT, USA) at 37 °C and 5 % CO_2_. No immortalization methods were used. Cell line authentication was performed using microsatellite analysis of Short Tandem Repeats (STRs), including a 10 markers/loci panel, by Genewiz Inc. (South Plainfield, NJ) (Available on request from authors).

### Genomic analyses – whole-exome sequencing and data analysis

DNA was extracted from the cell line samples using an AllPrep DNA/RNA Mini Kit (Qiagen), according to the manufacturer's protocol. DNA concentration was quantified using a Qubit dsDNA HS Assay (Thermo Fisher Scientific). About 0.5 μg of genomic DNA was fragmented by Hydrodynamic Shearing (Covaris, Inc.) to generate 150- to 280-bp fragments. After end repair, fragments were ligated to Illumina barcoded adapters, cleanup, amplified by PCR to enrich for adapter-ligated fragments, and controlled for quality. The library was then enriched by liquid-phase hybridization using Agilent SureSelect XT Human All Exon v6 (Agilent Technologies), following the manufacturer's recommendations. The captured library was amplified by PCR using indexing primers, cleanup, and quality assessment was done with the TapeStation 4200 (Agilent Technologies). Libraries are submitted to Illumina NovaSeq 6000 S4 PE100 (25 M reads). Sequence alignment and mutation calling were performed in Partek Flow Environment (Partek Inc). Sequence reads were aligned to the human genome hg38 build using bwa 0.7.2 [18]. Variants were called using Strelka 1.0.15 [19]. The called variants were annotated using the Annovar software [20]. Annotated calls were then filtered to show only protein-changing single-nucleotide variations (SNV) that were present at allele frequencies greater than 0.1 and coverage higher than 16 × . For copy-number aberration calling, data analysis was performed using Nexus Copy Number Discovery Edition Version 9.0 (BioDiscovery, Inc.). Samples were processed using the Nexus next-generation sequencing functionality with the FASST2 segmentation. The log ratio thresholds for single-copy gain and single-copy loss were set at +0.18 and −0.18, respectively. The log ratio thresholds for gain of two or more copies and for a homozygous loss were set at +0.6 and −1.0, respectively. Homologous repair deficiency (HRD) scores were derived from whole-exome sequencing data based on published algorithm by Sztupinszki e*t al.* [21].

### Immunohistochemistry analysis and western blot analyses of tumors

Immunohistochemistry analysis and interpretation for PAX8, p53, keratin (AE1/3), desmin, myogenin, MLH1, PMS2, MSH2 and MSH6 was performed on whole tissue sections as described in Supplementary Materials and Methods. For wester blot studies, tumour lysates were prepared as previously described (Supplementary Material and Methods) [22].

### *In vivo* PDX FGFR1 inhibitor study

Actively growing PDX tumors (AB734, AB739 and AB740) were harvested and cut into small pieces of approximately 2 × 2 × 2 mm before implantation into immunocompromised female mice subcutaneously. At day 5 post-implantation, the mice were randomly assigned to 2 experimental arms with 8 mice per arm. Mice in the treatment group received daily intraperitoneal (*i.p.)* injection of AZD4547 (Catalog No.S2801, Selleck Chemicals LLC, Houston, TX, USA) at 25 mg/kg for the duration of the study (22 days for AB739, 36 days for AB740 and 41 days for AB734) with the mice in the control group receiving daily *i.p.* injection of vehicle solution containing DMSO and Kolliphor. Tumor growth was monitored twice per week over the treatment period. The experiment was terminated when the subcutaneous tumor in the treatment and/or control group reached 15 mm in one of the dimensions. On the last day of the treatment, the mice were euthanized and necropsy was performed with the tumor harvested and weighed. The tumor as well as liver, lung and kidney tissues were submitted for histologic evaluation. All procedures were carried out according to the approved animal research ethics protocol AUP00002496 by the Animal Care and Use Committee at the University of Alberta.

## Results

### Clinicopathologic features of the study samples and experimental models

A total of 8 fresh tumor samples of gynecologic carcinosarcoma (7 uterine and 1 ovarian) were collected with attempts made to establish PDX tumor and cell line models. PDX tumor model development was successful in 6 cases (75 % success rate) with 5 uterine carcinosarcoma and 1 ovarian carcinosarcoma established beyond passage 3 (Table 1). All 6 PDX models were tumorigenic both as subrenal and subcutaneous implants, except AB778 (the ovarian carcinosarcoma PDX). A uterine carcinosarcoma cell line model (CL20) derived from a patient with FIGO stage IIIC1 disease was also established but the corresponding PDX tumor model ceased to grow past passage 1. The other uterine carcinosarcoma that failed to engraft as PDX tumor was derived from a patient with FIGO stage IA disease. The average time from implantation to the development of 1 cm^3^ growth ranged from 3 to 12 months in the initial passage (P1), and the rate of growth increased the subsequent generations, ranging from 1 to 4 months in the P2 to P3 generation. It is worth noting that there was sufficient tumor tissue collected for the 6 successfully established PDX tumor for concurrent attempts at cell line development but none were successful. All tumor sample were collected from the primary site (uterine or ovarian, respectively).

**Table 1:** The clinical and pathologic features of carcinosarcoma patient samples.

Case number	Model	Age	Presenting symptom	Primary tumor	Diagnosis	Percent sarcoma	Clinical stage	Surgery	Adjuvant treatment	Follow-up
AB734	PDX (P4)	68	PMB	Uterine	Carcinosarcoma (homologous)	5 %	IIIA (uterine serosal and bilateral adnexal involvement)	TAH-BSO + omentectomy	Carboplatin/paclitaxel + EBRT	NED at 38 months
AB739	PDX (P5)	60	PMB	Uterine	Carcinosarcoma with skeletal muscle differentiation	90 %	II	TAH-BSO + pelvic lymphadenectomy	Carboplatin/paclitaxel + EBRT	Vaginal vault recurrence at 21 months, resistant to carboplatin/paclitaxel, switched to Calyx but disease continued to progress, DOD at 33 months
AB740	PDX (P6)	78	PMB	Uterine	Carcinosarcoma with cartilaginous differentiation	95 %	IIIC1 (positive pelvic nodes, vaginal drop metastasis)	TAH-BSO + pelvic lymphadenectomy + omentectomy	Carboplatin/gemcitabine	Vaginal vault recurrence/progression during chemo at 2 months post-surgery, treated with adjuvant brachytherapy with complete response, NED at 48 months
AB768	PDX (P4)	80	PMB	Uterine	Carcinosarcoma with cartilaginous differentiation	50 %	IA	TAH-BSO + pelvic lymphadenectomy + omentectomy	Carboplatin/paclitaxel + EBRT	LFU at 12 months
AB778	PDX (P4)	68	Abdominal fullness and discomfort	Ovarian	Carcinosarcoma with focal cartilaginous differentiation; associated STIC	40 %	IV (omental and peritoneal metastasis)	TAH-BSO, omentectomy, peritoneal biopsy	Carboplatin/paclitaxel/bevacizumab	Initially good response to chemotherapy but disease progression at 4 months post-op, DOD at 10 months post-op (HPC germline testing showed no pathogenic BRCA1/2 mutations)
AB782	PDX (P6)	73	PMB	Uterine	Carcinosarcoma with skeletal muscle and focal cartilaginous differentiation	90 %	IA	TAH-BSO + pelvic lymphadenectomy	None	LFU after 3 months
CL20	Cell line (P20)	76	PMB	Uterine	Carcinosarcoma (homologous)	20 %	IIIC1 (positive pelvic nodes)	TAH-BSO + pelvic lymphadenectomy + omentectomy	Carboplatin/paclitaxel + EBRT	NED at 48 months

PDX: patient derived xenograft; PMB: postmenopausal bleeding; STIC: serous tubal intraepithelial carcinoma; TAH-BSO: total hysterectomy and bilateral salpingo-oophorectomy; EBRT: external beam radiation therapy; NED: no evidence of disease; DOD: died of disease; LFU: lost to follow-up.

The clinical and pathologic features of the 6 PDX and 1 cell line models are summarized in Table 1. All patients were post-menopausal with the age at initial diagnosis ranging from 60 to 80 years. All 6 patients with uterine carcinosarcoma (cases 1–6) presented with post-menopausal bleeding with the diagnosis of carcinosarcoma rendered on pre-operative endometrial biopsy. The clinical tumor stage at initial presentation ranged from organ-confined FIGO stage I-II (*n* = 3) to more advanced stages, with stage IIIA (bilateral adnexal involvement) in 1 case, stage IIIC1 (pelvic lymph node metastasis) in 2 cases and stage IV (diffuse peritoneal/omental involvement) in 1 case. Histologically, the carcinomatous components of all 6 uterine carcinosarcomas appear to be serous in type; 3 of 6 uterine carcinosarcomas contained prominent sarcomatous component that constitutes more than 90 % of the tumor while the remaining 2 uterine carcinosarcomas were comprised of an even admixture of carcinomatous and sarcomatous components. Heterologous sarcomatous differentiation was observed in 4 of the 6 uterine carcinosarcoma (2 with cartilaginous differentiation, 1 with skeletal muscle differentiation and 1 with mixed cartilaginous and skeletal muscle differentiation). AB778 was derived from a patient with ovarian carcinosarcoma that arose in association with a serous tubal intraepithelial carcinoma (STIC). The carcinomatous component was high-grade serous in histotype and there was an intimate nearly even admixture of carcinomatous and sarcomatous components through out the tumor. Clinical tumor mutation analysis showed no evidence of pathogenic *BRCA1, BRCA2, RAD51D/C* or *PALB1* mutations. By immunohistochemistry (performed on the uterine/ovarian tumor, summarized in Table 2), all uterine/ovarian carcinosarcoma displayed mutation pattern p53 staining (5 with overexpression and 2 with absent pattern), with concordant staining pattern observed between corresponding carcinomatous and sarcomatous components (Table 2). All tumors showed intact mismatch repair protein expression (MLH1, MSH2, MSH6 and PMS2). The carcinomatous components in all 7 carcinosarcomas showed PAX8 nuclear expression in keeping with Mullerian epithelial differentiation. The sarcomatous components all showed diffuse and strong vimentin expression. The two carcinosarcomas showing heterologous skeletal muscle differentiation showed focal desmin and myogenin expression.

**Table 2:** The histologic and immunophenotypic features of the six established carcinosarcoma patient-derived xenograft tumor models compared to the parental uterine/ovarian tumor samples.

**Case number**	**Tumor**	**Histology**	**Immunohistochemistry**			**Passage time *in vivo***
			***p53***	***MMR***	***Differentiation markers***	
AB734	Uterine	Carcinoma-predominant (95 %) carcinosarcoma	Mutated (OE)	Normal	CK7 and PAX8 expression with negative ER expression in carcinoma component	
	PDX (P3)	Carcinoma component only	Mutated (OE)	Normal	CK7 and PAX8 expression with negative ER expression	∼ 1.5 months
AB739	Uterine	Sarcoma-predominant (90 %) carcinosarcoma with skeletal muscle differentiation	Mutated (OE)	Normal	Focal desmin and myogenin expression	
	PDX (P3)	Sarcoma component only with skeletal muscle differentiation	Mutated (OE)	Normal	Focal desmin and myogenin expression	∼ 1 month
AB740	Uterine	Sarcoma-predominant (95 %) carcinosarcoma with cartilaginous differentiation	Mutated (A)	Normal	Focal S100 expression in chondroid foci	
	PDX (P3)	Sarcoma-predominant (90 %) carcinosarcoma with cartilaginous differentiation	Mutated (A)	Normal	Focal S100 expression in chondroid foci	∼ 1 month
AB768	Uterine	Carcinosarcoma with cartilaginous differentiation	Mutated (OE)	Normal	CK7 and PAX8 expression with negative ER expression in carcinoma component	
	PDX (P3)	Carcinoma component only	Mutated (OE)	Normal	CK7 and PAX8 expression with negative ER expression	2–3 months
AB778	Ovarian	Carcinosarcoma with focal cartilaginous differentiation	Mutated (A)	Normal	WT1 and PAX8 expression in carcinoma component	
	PDX (P3)	Carcinosarcoma	Mutated (A)	Normal	WT1 and PAX8 expression in carcinoma component	2–3 months
AB782	Uterine	Sarcoma-predominant (90 %) carcinosarcoma with skeletal muscle and focal cartilaginous differentiation	Mutated (OE)	Normal	Focal desmin and myogenin expression	
	PDX (P3)	Sarcoma component only with skeletal muscle differentiation	Mutated (OE)	Normal	Focal desmin and myogenin expression	∼ 1.5 month

PDX: patient derived xenograft; OE: p53 over-expression; A: absent p53 expression.

### Preservation of histologic, immunophenotypic and genetic features in patient derived xenograft models

**Fig. 1:**
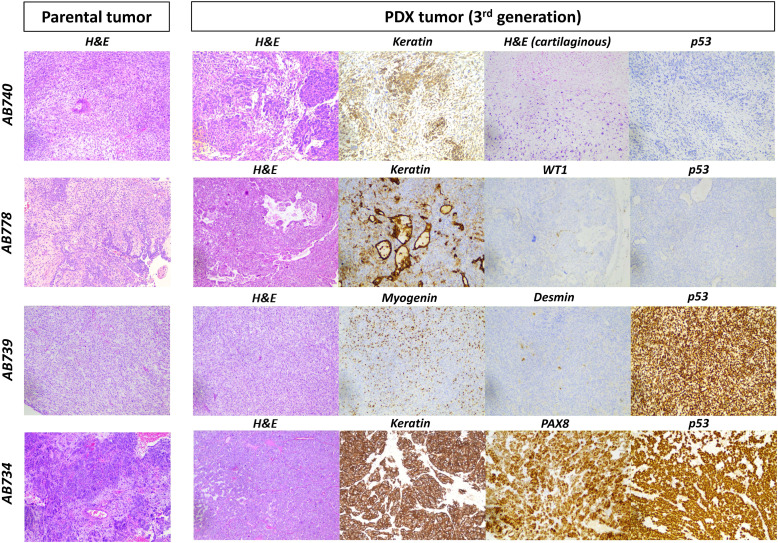
**Carcinosarcoma patient-derived xenograft (PDX) tumors exhibited the predominant histologic and immunophenotypic profiles as seen in the parental tumors.** In AB740, the parental uterine tumor was comprised predominantly of the sarcomatous element (90 %) with focal cartilaginous differentiation and shows a mutated (absent expression) p53 staining profile by immunohistochemistry, and the sarcomatous histology with focal keratin expression and the same mutated p53 staining pattern were preserved in the PDX model. In AB778, the parental ovarian tumor was comprised of a mix of carcinomatous (WT1-positive) and sarcomatous elements that both were p53 mutated (absent expression) by immunohistochemistry, and this admixture of WT1-positive carcinomatous and sarcomatous elements with the same mutated p53 staining pattern was preserved in the PDX model. In AB739, the parental uterine tumor was comprised predominantly of the sarcomatous element (90 %) with skeletal muscle differentiation (myogenin and desmin-positive), and the sarcomatous histology and the presence of skeletal muscle differentiation demonstrated by diffuse myogenin and focal desmin expression as well as a mutated (over-expression) p53 immunostaining pattern were preserved in the PDX model. In AB734, the parental uterine tumor was carcinoma-predominant (95 %) which was positive for keratin and PAX8 with mutated (over-expression mutation profile by immunohistochemistry, and the carcinomatous element with keratin and PAX8 expression as well as the same mutated p53 profile were preserved in the PDX model.

The histologic and immunophenotypic features of the 6 established PDX models are summarised in Table 2 and illustrated in Fig. 1. In two carcinosarcomas (1 uterine and 1 ovarian), the passaged PDX tumors maintained the same biphasic histology as seen in the parental tumor that showed an even admixture of carcinomatous and sarcomatous components (Fig. 1). Two of the 4 remaining PDX tumors were comprised exclusively of the sarcomatous component. The sarcomatous components exhibited the same heterologous differentiation (cartilaginous or skeletal muscle) as seen in the sarcomatous components of the corresponding parental tumor (Fig. 1). The remaining 2 PDX tumors displayed only carcinomatous differentiation, both of which were derived from uterine carcinosarcomas that were predominantly carcinomatous. By immunohistochemistry, all PDX tumors exhibited identical immunoprofile as that seen in the parental tumors (Table 2), with identical pattern of mutated p53 expression and intact MMR protein expression by immunohistochemistry.

**Fig. 2:**
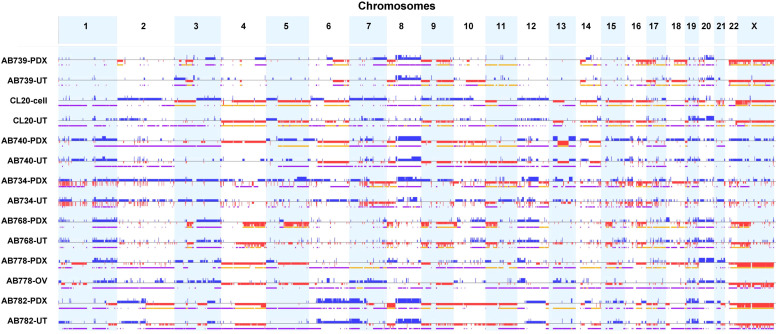
**Carcinosarcoma patient-derived xenograft (PDX) tumors exhibited similar genome-wide copy number profiles compared to parental tumors.** The six established carcinosarcoma PDX models all possessed a high degree of copy number alterations involving most of the chromosomes and these copy number alterations were generally preserved in the passaged PDX tumors (passage number 3). UT: uterine; OV: Ovarian.

By whole-exome sequencing analysis, all 6 PDX tumor models (at P2 passage) and 1 cell line model (passage 10) exhibited largely identical pattern of copy number variation (CNV) compared to the corresponding parental patient tumor samples (Fig. 2). All tumors showed a high degree of copy number alterations (CNA). The mutation profiles (SNV/indels) were also maintained in the PDX tumor and cell line models (Supplementary Table S1). For instance, among the 5 uterine carcinosarcomas showing over-expression p53 mutation staining patterns in the parental tumor samples (as described in Table 2), the PDX/cell line tumors harbored the same inactivating *TP53* mutations as identified in the parental tumors. The remaining 2 carcinosarcomas harbored the same frameshift or deletion mutation in *TP53* in both the parental and the PDX tumors that corresponded to the absent expression mutation pattern of p53 staining seen in the parental tumor samples. These phenotypic and molecular findings confirm that the developed PDX and cell line models were representative of the corresponding tumors from the respective patients. One case (AB734) harbored a somatic likely oncogenic *PPP2R1A* mutation (c.767C>*T*:exon6:p.S256F) that was present in both the PDX tumor and parental tumor samples, and another case (AB739) harbored a somatic likely oncogenic *FBXW7* mutation (c.1513C>*T*:exon12:p.R505C) that was present in both the PDX tumor and parental tumor samples. None of the parental, PDX or cell line samples analyzed harbored detectable oncogenic or likely oncogenic alterations *involving BRCA1, BRCA2, PALB2, MSH2, MSH6, MLH1* and *PMS2* or exonuclease domain mutations involving *POLE*.

### Molecular characterization informing potential therapeutic targets in gynecologic carcinosarcoma

To identify potentially targetable oncogenic alterations, we focused our analyses on targetable receptor tyrosine kinase (RTK) alterations, including known activating kinase SNV/indel mutations, genetic amplification/copy number gain and genetic fusion. The results are summarized in Table 3 and Supplementary Table S1**.** No known activating RTK SNV/indel mutations or fusions were identified. Among the established models, copy number gains, typically focal in nature, were frequently observed across multiple RTKs with the amplified RTKs typically present in both the parental and PDX tumors (Table 3). These included high level amplification (≥ 6 copies) of *FGFR1* in 2 cases (AB740 and CL20), NTRK1 in 1 case (AB734), *ERBB2* in 1 case (AB734). The latter tumor also showed diffuse and strong Her-2/neu expression with 3+ positivity in both the parental and third passage PDX tumors (data not shown).

**Table 3:** Receptor tyrosine kinases showing recurrent and/or high level copy number gains in the carcinosarcoma parental tumors and patient-derived xenograft (PDX)/cell line (CL) models. UT: uterine; OV: ovarian.

**Receptor Tyrosine Kinases**	**AB734**		**AB739**		**AB740**		**AB768**		**AB778**		**AB782**		**CL20**	
	***UT***	***PDX***	***UT***	***PDX***	***UT***	***PDX***	***Ut***	***PDX***	***OV***	***PDX***	***UT***	***PDX***	***UT***	***CL***
*ALK*	4	4									4	4	4	4
*AXL*	4	4			4	4			4	4	4	4	4	
*CSFR1*		6											4	4
*DDR2*	4				4	4	4	4	4	6				
*EGFR*											4	4	4	4
*EPHA2*	4	4			4	4	4	4			4		4	
*EPHB2*	4	4			5	4					4			
*EPHB3*	4	4					4	4	4	4				4
*ERBB2*	6	6												
*ERBB3*	4	5					5	5			4	4	4	4
*FGFR1*			5	5	8	8				4	5	5	6	6
*FGFR2*							4	5						
*FGFR3*		4			7		4	4						
*FGFR4*					4	4								
*FLT1*	4	6			5	6								
*FLT3*	4	6			5	6								
*FLT4*			4	4	4	4								
*MERTK*	4	5								4	5			
*MET*									4				4	4
*NTRK1*	6	6				4	4	4	4	5			5	5
*NTRK3*					4	4					4	4		
*PDGFRB*		6					5				4		4	4
*PTK7*	4	4					4	4			4	4		
*RET*													4	4
*ROS1*	4										5	5		
*RYK*	4	4					4	4		4				4
*STYK1*							4	4	4	4				

We also evaluated homologous repair deficiency (HRD) scores based on sequenced data of the parental and PDX tumor/cell line samples [21]. The summary HRD scores as well as the component scores for long region loss of heterozygosity (LOH), telomeric allelic imbalances (TAI), and large-scale transitions (LST) are shown in Supplementary Table S2. Among the 6 PDX models, 2 (1 ovarian and 1 uterine) showed HRD > 42 in both the parental and the PDX tumors. Three uterine carcinosarcoma models showed discordant HRD scores with scores of > 42 in the PDX models but below this threshold in the parental tumor sample. Only 1 case was found to have a low HRD score, in both parental and PDX tumors. The single established uterine carcinosarcoma cell line model showed HRD > 42 in both the parental and the cell line samples.

### Targeting FGFR1 copy number gain and protein over-expression with FGFR1 small molecule inhibitor

**Fig. 3:**
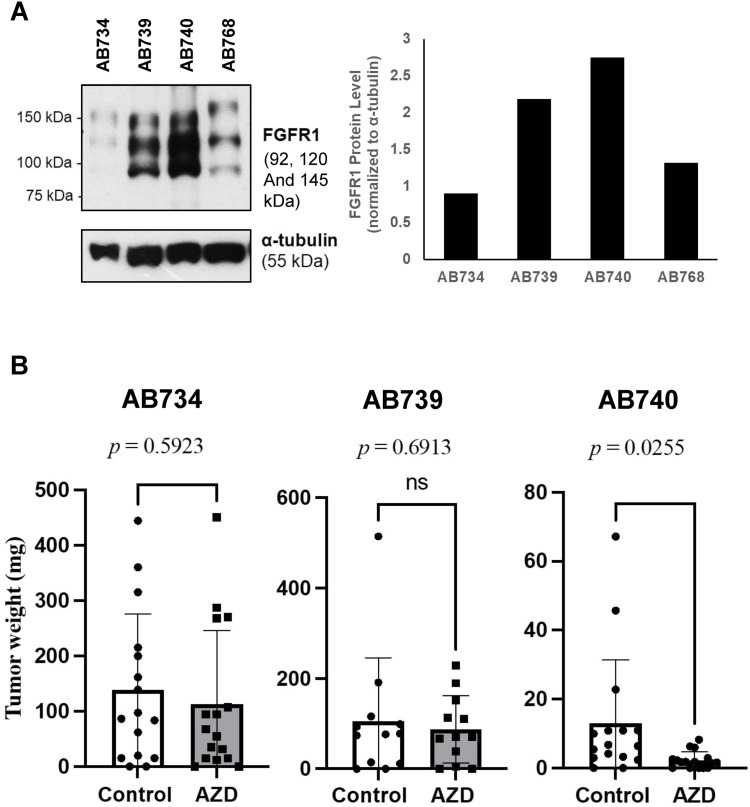
**FGFR1 expression in uterine carcinosarcoma patient-derived xenograft (PDX) tumors and *in vivo* response to small molecular inhibitor of FGFR1 inhibitor.** A). FGFR1 western blot analysis and the corresponding densitometry graph showed greater FGFR1 protein expression by *FGFR1*-amplified uterine carcinosarcomas (AB739 and AB740) compared to non-amplified uterine carcinosarcomas (AB734 and AB768) (uncropped blots shown in Figure S1), B). The effects on *in vivo* tumor growth by FGFR1 small molecule inhibitor (AZD4547) on a FGFR1-highly-amplified (copy number of 8)/overexpressed uterine carcinosarcoma (AB740, 36 days treatment, *n* = 16 tumors for each group), a FGFR1 moderately amplified (copy number of 5) uterine carcinosarcoma (AB739, 22 days treatment, *n* = 16 tumors for each group) and a FGFR1 copy number neutral uterine carcinosarcoma (AB734, 41 days treatment, *n* = 16 tumors for each group).

Given the clinical efficacy observed in targeting ERBB2 amplification in the closely related p53-mutated endometrial serous carcinoma [23], we focused on the focal *FGFR1* copy number gain/amplification as a novel and potentially targetable molecular alteration in uterine carcinosarcoma. We evaluated FGFR1 expression in the established PDX tumor models by western blot and found markedly increased FGFR1 expression in AB740 which showed high level *FGFR1* amplification (8 copies) compared to the tumors without *FGFR1* copy number gain (Fig. 3A). Given that FGFR1 can auto-dimerize when over-expressed [24], we selected AB740, AB739 (lesser degree of FGFR1 copy number gain and moderate protein expression) and AB734 (with no copy number gain in FGFR1 and low protein expression but showing copy number gains involving multiple other RTKs) for *in vivo* evaluation using a small molecule inhibitor for FGFR1 - AZD4547. The treatment group received daily intraperitoneal injection of AZD4547 (25mg/kg) and the control group received DMSO/Kolliphor vehicle control. In AB740, AZD4547 resulted in a significant inhibition of tumor growth (tumor weight) (*p* = 0.026) as shown in Fig. 3B, while no significant difference in tumor growth was observed between the treatment and the control groups in AB739 and AB734 (*p* = 0.69 and *p* = 0.59 respectively). There was no significant difference in mouse body weights between the treatment and the control groups in these studies with no grossly or histologic apparent tissue damages to the liver, lungs or kidneys at necropsy.

## Discussion

Gynecologic carcinosarcomas are rare and the benefit of chemotherapy is poorly understood, there are only a limited number of targeted therapies available clinically or under clinical evaluation [25]. These include Poly (ADP-ribose) polymerase (PARP) inhibitor for *BRCA1/2*-mutated or HRD ovarian carcinosarcoma [[26], [27], [28]], with evidence extrapolated from high-grade tubo-ovarian serous carcinoma and anti-Her-2 therapy to *ERBB2*/Her-2 amplified uterine carcinosarcoma, which is under clinical trial evaluation (NCT05256225) [29]. These targetable molecular alterations exist however in only a subset of ovarian and uterine carcinosarcomas. As such, continued efforts in identifying and validating additional therapeutic vulnerabilities of gynecologic carcinosarcoma in the preclinical setting are much needed. Several groups have demonstrated successful development of PDX models of gynecologic carcinosarcoma; presently, there are 9 established uterine carcinosarcoma and 1 established ovarian carcinosarcoma PDX models reported in the literature [[12], [13], [14], [15], [16]]. However, only half of the PDX models have been molecularly annotated in a comprehensive manner at whole genome or whole exome levels to better inform potential therapeutic opportunities. Given the known genetic diversity of uterine and ovarian carcinosarcomas, we set out to establish more molecularly annotated carcinosarcoma PDX models and have successfully established 6 PDX models (5 uterine and 1 ovarian carcinosarcoma) with an overall success rate of 75 %, compared to an engraftment rate that ranged from 29 % to 75 % in prior published series [12,14,30]. Interestingly, we were unable to establish concurrent cell line model among the 6 cases with successful PDX model development. Cell line model development was successful in a seventh case but the corresponding PDX model development failed as it could not be passaged beyond its first generation, nor were a subsequent attempt successful at cell line derived xenograft (CDX) model development. This raises the possibility that the development condition of different experimental models including PDX models may preferentially select for certain molecular and biologic subset of carcinosarcomas or even intra-tumoral subclone(s) of carcinosarcomas, and this underscores a limitation of PDX and cell line models that must be considered in therapeutic development.

With regards to the validity of these developed models, the propagated PDX models from all 5 uterine and 1 ovarian carcinosarcoma all exhibited histologic, immunophenotypic and molecular similarity to the corresponding parental tumors. All 5 uterine PDX models exhibited a *TP53*-mutated and MMR-proficient (MMRp) molecular landscape with a high degree of CNA and would be classified under the *p53 abnormal* (copy number high) molecular subgroup based on TCGA endometrial cancer molecular classification. The single ovarian carcinosarcoma PDX exhibited a combination of histologic, immunophenotypic and molecular features that indicate an origin from tubal high-grade serous carcinoma, given the association with STIC. Histologically, while a biphasic carcinosarcoma histology was only captured and preserved in 2 of the 6 PDX models, the predominant component from the remaining 4 carcinosarcomas were captured and preserved by the corresponding PDX models. Interestingly, the PDX tumor recapitulated the same lineage of heterologous sarcomatous differentiation as seen in the corresponding parental tumor. Given our understanding that gynecologic carcinosarcomas are carcinomas that underwent sarcomatous transdifferentiation (sarcomatoid/metaplastic carcinoma), this observation indicates that the exact lineage of mesenchymal transdifferentiation (sarcomatous transformation) is regulated by the tumor cells. As the sarcomatous component of carcinosarcoma can display a range of mesenchymal phenotype that ranges from an undifferentiated state (homologous) to a variably differentiated mesenchymal state along skeletal muscle, cartilage, osseous and adipocytic line of differentiation, our observation here suggests that it is not a stochastic/random process at the tumor cellular level but rather a controlled/programed process that commit the sarcomatous transdifferentiated tumor cells to a specific line of mesenchymal cell differentiation.

Another noteworthy observation is that while most gynecologic carcinosarcomas including all 6 PDX tumors here exhibit a high degree of copy number, the passaged PDX tumors retained the CNV profiles after several hundred cycles of proliferation from the parental tumor seeds to PDX passage 2 tumors. This suggests either that the initial event(s) that resulted in the chromosomal structural instability was short-lived or that this evolved state of chromosomal structural instability embeds molecular alterations that confer growth/survival advantage (*i.e.* period of stasis in a punctuated equilibrium model) [31]. On that note, focal genomic copy number gain/amplification in RTKs such as *FGFR1* and *ERBB2* identified in the PDX models here were conserved through successive *in vivo* passaging, which suggests that it may confer growth/survival advantage. This has been demonstrated to be the case in a closely related uterine cancer type – serous carcinomas (usually *TP53*-mutated with a high degree of CNA), where *ERBB2* amplification/copy number gain contributes to tumor growth which can be targeted to improve patient survival clinically [23]. A more recent clinical study using an optimized HER2-targeted antibody-drug conjugate (trastuzumab deruxtecan) in patients with HER2 (encoded by *ERBB2*) immunohistochemistry high (2+ or 3+) and low (1+) uterine carcinosarcoma found clinical efficacy in both HER2-high and -low groups [32]. Given the precedence in targeting *ERBB2-*amplified/HER2-expressing carcinosarcoma, we focused our attention on *FGFR1* copy number gain/amplification as another potentially targetable alteration in carcinosarcoma.

FGFR1 is a member of the fibroblast growth factor receptor family that includes four receptor tyrosine kinases - FGFR1–4 [[33], [34], [35]]. Like other receptor tyrosine kinases, FGFR1 consists of an extracellular immunoglobulin-like ligand binding domain, a transmembrane domain and an intracellular tyrosine kinase domain, which upon activation by fibroblast growth factor (FGF) can result in the activation of ERK/MAPK pathway. These receptors can also activate through auto-dimerization in the absence of ligand stimulation [24]. Activating mutations of FGFR1–4 occur in a variety of human cancer and they can involve activating point mutations (SNV), genetic fusion and genetic amplification [[33], [34], [35]]. In contrast to FGFR2–3, activating SNV mutation and genetic fusion involving FGFR1 occurs infrequently [36]. The most common genetic alteration involving FGFR1 is copy number gain, which is known to occur in a significant subset of breast cancer, non-small cell lung cancer and urothelial cancer. In uterine carcinosarcomas, *FGFR1* genomic amplification appear to occur in about 10 % of cases based on c-Bioportal data [37]. Among the previously reported PDX models of uterine carcinosarcomas with available CNV data, *FGFR1* genomic amplification was identified in 1 of 5 tumors [12]. However, it is important to note that amplification involving *FGFR1* does not necessarily reflect protein overexpression, which is required for oncogenic activity. In our present cohort of 6 gynecologic carcinosarcoma PDX models, AB740 demonstrated high copy number gain (8 copies) of *FGFR1* with the highest FGFR1 protein expression by immunoblot analysis compared to the other tumors. The presence of amplification-related overexpression suggests a potential oncogenic role for FGFR1. Currently, there are several FGFR-targeted therapies under phase 1–3 clinical evaluation with a number of inhibitors approved for clinical use [36]. These include small molecule tyrosine kinase inhibitors that are selective for FGFR1–4, such as AZD4547. Based on the existing experience in using small molecule inhibitor to target FGFR1 and other RTKs, they are typically most effective in targeting tumors harboring activating kinase domain mutation or genetic fusion, whereas their efficacy in targeting genetic amplification is variable and suboptimal in general [38]. However in AB740 which showed high copy number gain (8 copies) and high protein expression of FGFR1, we observed a significant inhibition of tumor growth *in vivo*. In contrast, in carcinosarcoma PDX tumors with moderate or no copy number gain in FGFR1 and lower protein expression, AZD4547 did not cause a significant reduction in tumor growth The lack of significant growth inhibition seen in AB739 showing lower level FGFR1 copy number gain (5 copies) and lower FGFR1 protein expression than AB740 indicate that significant oncogenic FGFR1 activation (presumably through self-dimerization) may require a higher level of protein expression. Overall, the *in vivo* findings suggest an oncogenic role for highly amplified and highly expressed FGFR1 in uterine carcinosarcoma. Aside from small molecule inhibitor, an alternative and a likely more effective approach is to use antibody-drug conjugate targeting amplified/over-expressed FGFR1 [39], which may offer clinical efficacy even in tumors expressing FGFR1 with low to moderate copy number gain (such as AB739). Future study is needed to evaluate the effectiveness of such antibody-based therapeutic approach.

Another potential therapeutic opportunity highlighted by HRD score analysis is that 5 of 6 uterine carcinosarcoma models (4 of 5 PDX and 1 of 1 cell line) and the one ovarian carcinosarcoma PDX model showed high HRD score (> 42). It is important to note that there is no established HRD cut-off for uterine carcinosarcomas or endometrial carcinomas at present in the context of a therapeutic prediction cut-off for PARP inhibitor. The cut-off of 42 was extrapolated based on the cut-off used for ovarian carcinoma. The finding of high HRD score in uterine or ovarian carcinosarcoma is not unexpected as high HRD score is known to occur in a significant subset of *TP53*-mutated high-grade endometrial carcinoma/carcinosarcoma and in high-grade tubo-ovarian serous carcinoma/carcinosarcoma [40,41], with PARP inhibitor therapy being utilized clinically for the latter. In a recently reported series of 3 established uterine carcinosarcoma PDX [12], 2 of 3 PDX tumors in the latter passages showed high HRD score, though all 3 showed significant tumor growth inhibition with single agent PARP inhibitor treatment compared to vehicle control. While the predictive role of HRD score for PARP inhibitor in uterine carcinosarcoma remains unclear, PARP inhibitor does warrant further therapeutic exploration for *TP53*-mutated (copy number-high) molecular type of uterine carcinosarcoma.

In summary, we have successfully established, validated and molecularly annotated 6 PDX models and 1 cell line model of gynecologic carcinosarcoma and these models are available for sharing with the research community upon request. We have identified a number of molecular alterations that may be targetable using compounds currently under clinical use or under clinical trial study, and demonstrated preclinical evidence in support of targeting amplified/over-expressed FGFR1 in uterine carcinosarcoma. These established models that recapitulate the molecular complexity and diversity of gynecologic carcinosarcoma can therefore serve as valuable preclinical models to enable more efficient preclinical evaluation for targeted therapies in this era of precision gynecologic oncology.

## Data availability

The datasets generated during and/or analysed during the current study are available from the corresponding author on reasonable request.

## CRediT authorship contribution statement

**Nelson K.Y. Wong:** Conceptualization, Data curation, Investigation, Methodology, Project administration, Writing – original draft. **Marta Llaurado Fernandez:** Data curation, Methodology. **Hannah Kim:** Data curation, Investigation. **Pooja Praveen Kumar:** Investigation. **DuPreez Smith:** Investigation. **James Key:** Investigation. **Yen-Yi Lin:** Data curation, Formal analysis. **Stanislav Volik:** Data curation, Formal analysis. **An Nhien Tong:** Data curation. **Stephane Le Bihan:** Data curation, Formal analysis. **Colin C. Collins:** Methodology, Supervision. **Yangxin Fu:** Resources, Writing – review & editing. **Hui Xue:** Investigation. **YZ Wang:** Methodology, Supervision. **Martin Köbel:** Data curation, Investigation, Writing – review & editing. **Mark S. Carey:** Conceptualization, Funding acquisition, Resources, Supervision, Writing – original draft. **Cheng-Han Lee:** Conceptualization, Formal analysis, Resources, Supervision, Writing – original draft.

## Declaration of competing interest

The authors declare that they have no known competing financial interests or personal relationships that could have appeared to influence the work reported in this paper.

## Appendix Supplementary materials

Image, application 1
