# Supplementary material for: Establishment and characterization of preclinical models of human gynecologic tract carcinosarcomas demonstrates targetable FGFR1 alterations
Source: Transl Oncol. 2025 Nov 6;63:102591. doi: 10.1016/j.tranon.2025.102591 (PMC12639385; doi:10.1016/j.tranon.2025.102591)

**Supplementary Materials and Methods**

***Patient-derived xenograft* *(PDX) development***

Fresh or cryopreserved tumor was cut into multiple 4 × 2 × 1 mm pieces (12-24 pieces/specimen) and grafted under renal capsules of 3-6 NOD-*scid* IL2Rgamma^null^ or NOD-*Rag1^null^ IL2rg^null^* mice (1–2/kidney). Briefly, after the fur removal on the back of the recipient mouse and skin preparation with chlorhexidine and alcohol, an incision of approximately 1.0 cm was made along the midline of the skin in the back of an anesthetized mouse. Another incision of 0.6 to 0.8 cm was made on the muscle wall above the left ovary and kidney. The kidney was exteriorized by applying gentle pressure around the organ using forefingers and thumbs. Fine forceps were used to gently pinch and lift the capsule from the parenchyma of the kidney to facilitate the creation of a pocket using blunt dissection. The cut edge of the kidney capsule was lifted with a pair of fine forceps and the graft was inserted into the pocket under the capsule using a polished glass pipette. Two tumor pieces were inserted into each subrenal pocket, one on each side of the kidney. Once the grafting procedure was completed, the kidney was gently eased back into the body cavity, and the edges of the body wall were aligned and closed with the aid of an absorbable suture. The incision of the muscle wall and the subsequent procedures were repeated on the other side of the mouse for the implantation under the renal capsule of the right kidney. After the closing of the right muscle wall, the skin incision was closed with a non-absorbable suture. All procedures, including post-surgery monitoring, were carried out in accordance with protocol A18-0105 approved by the Animal Care Committee at the University of British Columbia. Xenografts were retrieved after it reached about 500-1000 mm^3^ in size with a portion utilized for histologic and molecular evaluation and the remaining tissue (passage #1) re-grafted to generate PDXs (passage #2). Part of the tumor was cryopreserved with 90% FBS/10% dimethyl sulfoxide for future engraftment. The PDXs were serially passaged in the subrenal site to passage #3, at which time the PDX tumor line was considered stable. The PDXs were then transferred to subcutaneous location and further passaged.

***Immunohistochemistry analysis of tumors***

Immunohistochemistry analysis and interpretation for PAX8, p53, keratin (AE1/3), desmin, myogenin, MLH1, PMS2, MSH2 and MSH6 was performed on whole tissue. For immunophenotype validation, cell differentiation markers such as PAX8, keratin, desmin and myogenin were performed on 2 tumor sections each for the parental and the passaged PDX tumors. P53, MLH1, PMS2, MSH2 and MSH6 were performed on a representative tumor section each for the parental and the passaged PDX tumors. For, PAX8, vimentin, p53, keratin (AE1/3), desmin, myogenin, MLH1, PMS2, MSH2 and MSH6 immunohistochemistry, the mouse monoclonal PAX8, clone MRQ-50 (catalog number: 363M) antibody was obtained from Biocare Medical (Concord, California, United States), with the mouse monoclonal vimentin, clone V9 (catalog number: GA63061-2), mouse monoclonal p53, clone DO-7 (catalog number: M7001) antibody, the mouse monoclonal cytokeratin, clone AE1/3 (catalog number: IR05361-2) antibody, the mouse monoclonal desmin, clone D33 (catalog number: IR60661-2) antibody, the mouse monoclonal myogenin, clone F5D (catalog number: IR06761-2) antibody, the mouse monoclonal MLH1, clone ES05 (catalog number: IR07961) antibody, the mouse monoclonal PMS2, clone EP51 (catalog number: IR08761) antibody, the mouse monoclonal MSH2, clone FE11 (catalog number: IR08561) antibody and the mouse monoclonal MSH6, clone EP49 (catalog number: IR08661) antibody all obtained from Dako (Burlington, Ontario, Canada). For PAX8, primary incubations were performed for 60 min (37°C) at 1:200 dilution using Ventana antibody diluents, followed by Universal Secondary Antibody for 32 min at 37°C and the detection system used was the Ventana OptiView 3,3′-Diaminobenzidine (DAB) kit. The other primary antibodies from Dako were ready-to-use antibody for autostainer and were performed according to manufacturer’s protocols using horseradish peroxidase (HRP)-based DAB detection system on Dako Omnis platform (Burlington, Ontario, Canada).

For MMR proteins, cases were scored as showing intact expression if any tumour cell nuclei showed nuclear staining and deficient if the tumour nuclei were unstained in the presence of internal positive control immunoreactivity. For PAX8, ER and p53, only nuclear staining was considered and evaluated. PAX8 and ER immunostains were scored as positive if any tumor cells exhibited moderate to strong positive (definite) nuclear staining. p53 immunostaining was considered to be aberrant (mutated/inactivated) if the tumor exhibited 1) diffuse moderate to strong uniform nuclear staining in ≥ 70% of the tumor cells (overexpression mutation pattern), 2) complete absence of nuclear staining in the tumor cells in the presence of focal nuclear staining of the stromal cells (absent expression mutation pattern) or 3) cytoplasmic p53 staining in tumor cells (cytoplasmic mutation pattern). p53 immunostaining was considered normal (wild type pattern) if any degree of non-diffuse nuclear staining (<70%) of the tumor cells was present. For cytokeratin and desmin, only membrane and cytoplasmic staining was evaluated and for myogenin, only nuclear staining as evaluated.

**Western blot analysis of tumors**

Briefly the tumour tissues (10-30 mg) were placed in 75-100uL of lysis buffer to tubes with ceramic beads and homogenized at 5000rpm for 20 seconds x 2, purified by centrifugation at > 10,000 rpm for 15 minutes at 4C after snap freezing the homogenate.  Proteins were then quantified using Pierce BCA Protein Assay Kit (Cat # 23225). Twenty micrograms of protein were loaded into each lane and separated into 8% SDS-PAGE gel and transferred to nitrocellulose membranes. The membrane was probed with FGFR1 (cell signaling Cat # 9740) or vinculin (Sigma V9131). Appropriate concentrations of horseradish peroxidase (HRP)-conjugated secondary antibodies (goat-anti-rabbit or goat-anti-mouse, Sigma A9917 and A0545) were used accordingly. Western blots were imaged using Immobilon Western Chemiluminescent HRP reagent (Sigma Millipore WBKLS0500) and developed by autoradiograph.

**Supplementary Table**

**Table S1: Summary of the somatic mutation profiles of the established carcinosarcoma PDX tumor/cell line models.** The oncogenic/likely oncogenic mutations, non-missense somatic mutations and receptor tyrosine kinase copy number gains displayed for each case were found in both the parental tumor and the passaged PDX tumor (passage #3)/cell line (* passage 10). All parental tumors and the corresponding passaged tumors/cell line harbored identical oncogenic/likely oncogenic mutations (Tier 1 and 2 mutations). Only Tier 3 somatic mutations with apparent pathogenic effects (including non-sense and frameshift mutation) shared by the parent tumor and the passaged tumor/cell line are illustrated here.

|  | **AB734** | **AB739** | **AB740** | **AB768** | **AB778** | **AB782** | **CL20** |
| --- | --- | --- | --- | --- | --- | --- | --- |
| ***Known Oncogenic/likely oncogenic mutations*** |  |  |  |  |  |  |  |
| ***TP53*** | **missense (p.R273H)** | **missense (p.S241F)** | **deletion/splice variant** | **missense (p.H179Y)** | **frame-shift (p.D281fs)** | **missense (p.E286Q)** | **missense (p.K132N)** |
| ***PPP2R1A*** | **missense (p.S256F)** |  |  |  |  |  |  |
| ***FBXW7*** |  | **missense (p.R505C)** |  |  |  |  |  |
| ***CAMTA1*** |  |  |  |  | **non-sense (p.G24X)** |  |  |
|  |  |  |  |  |  |  |  |
| ***Additional somatic mutations with apparent pathogenic effects*** |  |  |  |  |  |  |  |
| *AGO2* |  |  | p.E778X |  |  |  |  |
| *APOB* |  |  |  | p.S2928X |  |  |  |
| *BRWD1* |  | p.C208fs |  |  |  |  |  |
| *C6orf89* |  |  |  | p.Q54fs |  |  |  |
| *CADPS* |  |  |  |  |  | p.L1204fs |  |
| *CEP83* |  |  |  | p.L224fs |  |  |  |
| *CHST5* |  |  | p.W145X |  |  |  |  |
| *COL11A1* |  |  |  |  |  | p.Q1098X |  |
| *CPA1* | p.Q359X |  |  |  |  |  |  |
| *CTNND1* |  |  |  | p.Q827X |  |  |  |
| *DNTTIP2* |  |  |  |  |  | p.S148X |  |
| *ENO1* |  |  |  |  |  |  | p.E101fs |
| *EVC* |  |  |  |  |  |  | p.A203fs |
| *FRMPD4* | p.S1006X |  |  |  |  |  |  |
| *GABRB1* |  |  |  | p.K222X |  |  |  |
| *GATC* |  |  |  |  |  |  | p.G120fs |
| *GRIN2C* |  |  |  |  |  | p.Q674X |  |
| *IGFN1* |  |  |  |  |  | p.P23fs |  |
| *JOSD2* |  |  |  |  | p.E180del |  |  |
| *LRRC8D* |  |  |  | p.L176fs |  |  |  |
| *MGAM2* |  |  |  |  |  | p.S114X |  |
| *MST1L* |  |  | p.Q40fs |  |  |  |  |
| *PHOSPHO2* |  |  | p.D241_X242insY | |  |  |  |
| *PIK3R1* |  |  | p.I566_D569del |  |  |  |  |
| *RAX2* |  |  |  | p.E21X |  |  |  |
| *RRBP1* |  |  |  |  |  |  | p.Q1309X |
| *SYNE2* |  |  |  |  |  | p.E2033fs |  |
| *TLE1* | p.S42del |  |  |  |  |  |  |
| *TLR8* | p.E38X |  |  |  |  |  |  |
| *TMPPE* |  |  |  | p.T192fs |  |  |  |
| *ZIC1* |  |  |  |  |  |  | p.K311X |
| *ZNF449* |  |  |  |  |  |  | p.X519fs |
| *ZNF766* |  |  |  | p.V97X |  |  |  |

**Table S2:** Homologous repair deficiency (HRD) status of the carcinosarcoma parental tumors and patient-derived xenograft tumor (PDX)/cell line samples.

|  | **LOH** | **TAI** | **LST** | **HRD score** |
| --- | --- | --- | --- | --- |
| AB734 (parental uterine tumor) | 6 | 13 | 8 | 27 |
| AB734 (PDX tumor) | 8 | 19 | 20 | 47 |
| AB739 (parental uterine tumor) | 14 | 15 | 12 | 41 |
| AB739 (PDX tumor) | 16 | 17 | 15 | 48 |
| AB740 (parental uterine tumor) | 8 | 15 | 11 | 34 |
| AB740 (PDX tumor) | 11 | 14 | 12 | 37 |
| AB768 (parental uterine tumor) | 11 | 16 | 8 | 35 |
| AB768 (PDX tumor) | 12 | 20 | 13 | 45 |
| AB778 (parental ovarian tumor) | 8 | 18 | 21 | 47 |
| AB778 (PDX tumor) | 10 | 19 | 18 | 47 |
| AB782 (parental uterine tumor) | 13 | 21 | 16 | 50 |
| AB782 (PDX tumor) | 13 | 22 | 17 | 52 |
| CL20 (parental uterine tumor) | 16 | 26 | 13 | 55 |
| CL20 (cell line) | 18 | 22 | 10 | 50 |

PDX: patient derived xenograft; LOH: Long regions of loss of heterozygosity; TAI: Telomeric allele imbalances; LST: Large-scale transitions.

**Supplementary Figure**

**Figure S1:** Uncropped original blot for Figure 3A. NA: not applicable samples unrelated to the study


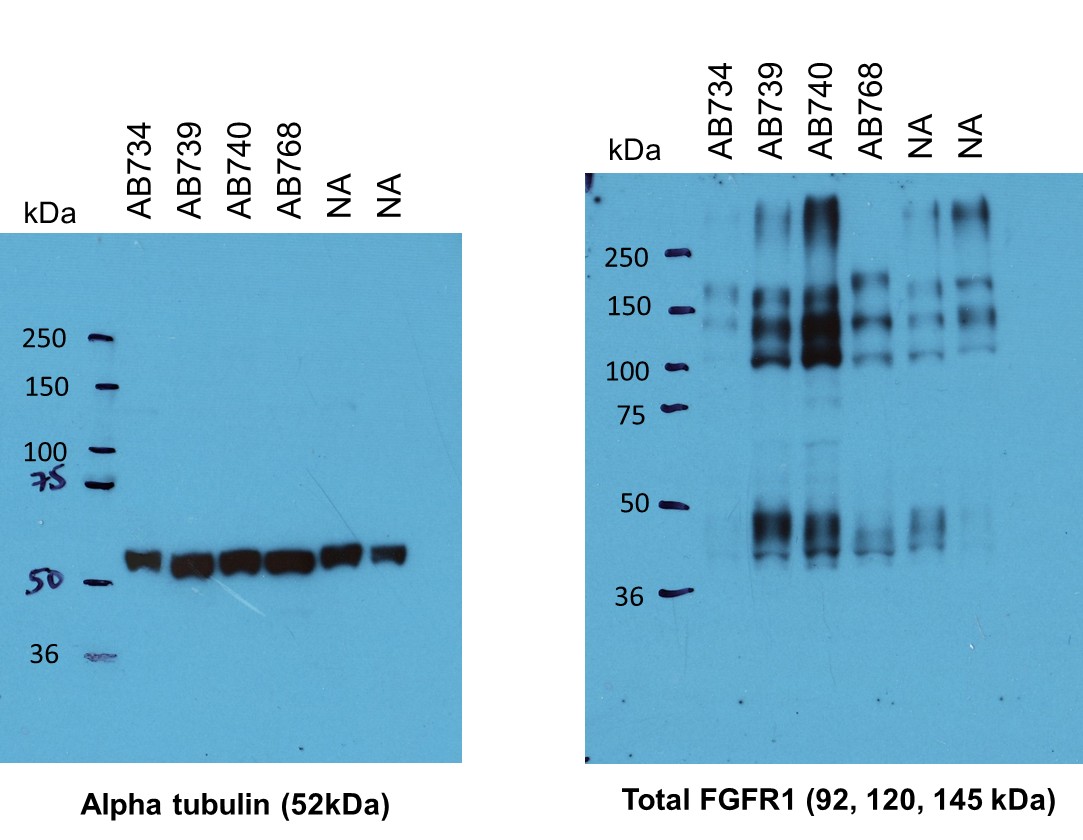

Supplement: Supplementary file 1 [file mmc1.docx]
